# A retrospective longitudinal analysis of risk factors, treatment outcomes and imaging parameters of ventricular collapse in idiopathic intracranial hypertension

**DOI:** 10.1186/s12987-025-00717-x

**Published:** 2025-11-05

**Authors:** Riccardo Serra, Smruti Mahapatra, Sakibul Huq, Michael Meggyesy, Noah Leviton Gorelick, Lacie Manthripragada, Abhay Moghekar, Mark Gregory Luciano

**Affiliations:** 1 Department of Neurosurgery, University of Maryland School of Medicine, 22 St Greene Street, Baltimore, 21201 MD USA; 2 Department of Neurosurgery, Johns Hopkins University School of Medicine, Baltimore, MD USA; 3 Department of Biomedical Engineering, Johns Hopkins University Whiting School of Engineering, Baltimore, MD USA; 4 Department of Neurology, Johns Hopkins University School of Medicine, Baltimore, MD USA

**Keywords:** Ventricular collapse, Idiopathic intracranial hypertension, Pseudotumor cerebri, Slit-ventricle syndrome

## Abstract

**Objective:**

Ventricular collapse is a prevalent yet poorly understood complication of ventriculo-peritoneal shunting (VPS) in idiopathic intracranial hypertension (IIH). By identifying the risk factors of ventricular collapse (VC), this study aims to characterize the clinical progression and treatment of IIH and its complications. The relationships between ventricular area, symptoms and treatments were assessed longitudinally with ventricular segmentation on MRI/CT imaging, and correlated with other risk factors of IIH and VC.

**Methods:**

We retrospectively reviewed 147 patients who underwent VPS for IIH at our Institution, and identified 73 shunt-naïve subjects. Manual segmentation of CT/MRI scans was performed at each clinical stage (baseline, post-shunting, post-collapse and after each intervention). Variables collected included valve type and opening-pressure, shunt revisions, use of anti-siphoning devices (ASD), comorbidities, venous sinus hypoplasia/stenosis, stenting, general demographics. Linear univariate regression models were used to determine the association between individual risk factors and VC, and quantitatively assess the effect of treatment. Two multivariate models were tested, including Pre-Shunting and Post-Shunting variables, to quantify their association with VC.

**Results:**

Of 73 IIH patients with new shunts, 32 experienced collapse (uni- or bilateral, 26.5% of the total). In shunt-naïve patients, collapse was associated with pre-shunting (rho = −0.36; *p* = 0.001) and post-shunting ventricular area (rho = 0.62; *p* = 0.0002). Both collapse and ventricular area were correlated with shunt-related symptoms at 6 months (rho = −0.29; *p* = 0.01). Shunt adjustment, addition of ASDs, valve replacement proved to be effective strategies to re-expand the ventricles and reduce symptoms. Nonetheless, a significant fraction of patients remained symptomatic after multiple treatments, suggesting a complex etiology for VC. On univariate analysis, catheter revisions were more common in the VC group, while the multivariate model with Post-Shunting factors was significantly associated with VC.

**Conclusions:**

In newly VP-shunted IIH patients, small ventricular area predisposes to collapse and headaches, while higher valve settings and ASDs may reduce the risk of collapse and promote symptomatic improvement. Within the restraints of a retrospective analysis, this study is the first to analyze the risk factors of VC in IIH patients, longitudinally integrating the clinical progression with ventricular imaging. Further studies are warranted to better understand the clinical progression of collapse.

**Supplementary Information:**

The online version contains supplementary material available at 10.1186/s12987-025-00717-x.

## Background

Idiopathic intracranial hypertension (IIH) is a poorly understood disease defined by increased intracranial pressure (ICP) in absence of other known causes and normal CSF [1]. The typical presentation includes headaches, papilledema, transient visual obscurations and blindness [2], as well as abducens nerve palsy, tinnitus, and mood disturbance [2]. Acetazolamide and topiramate have been mainstays of treatment [1], though over the past decades CSF diversion surgery (i.e. ventriculo-peritoneal shunting (VPS)) has grown in popularity [3–12]. While VPS is efficacious in reducing symptoms and preserving vision, treatment frequently fails or gives rise to major complications, such as headaches, confusion, changes in vision and dizziness [13]. Current guidelines recommend CSF diversion in patients experiencing rapid vision deterioration, persistent/worsening headaches, or intolerance to medical treatments [8].

One of the most troublesome complications of CSF diversion in IIH is ventricular collapse (VC) [13]. While the etiology is unknown, it is thought that the slit-like appearance of ventricles in IIH and elevated ICP may predispose them to collapse upon VPS [14–16]. Uni- or bilateral VC has been described as part of the Slit-Ventricle Syndrome (SVS) when diagnosed in association with headaches and slow valve-refill [17–19]. Interestingly, little is known of the similarities between VC in IIH and some features of SVS, limiting our understanding of shunt-related symptomatology. Despite seeing a general improvement after shunting, these patients worsen progressively in the months post-surgery as a consequence of overdrainage and ventricular collapse, with significant costs [19, 20]. Intermittent and supraphysiologic shunting, small ventricles and raised ICP, as well as depression, obesity and anxiety have all been reported as possible explanations for headaches, but their impact has never been fully understood and characterized [19].

The primary objective of this study was to determine the incidence and risk factors of VC in shunted patients with IIH, to define the patterns of ventricular shrinkage and shape after CSF diversion, to study its clinical progression, and to describe therapeutic strategies to address its symptomatology. Furthermore, we analyzed the effect of individual risk factors (obesity, venous sinus stenosis/stenting, hardware used) on ventricular collapse and area, and correlated them with the clinical presentation by means of uni- and multivariate statistical models. Manual segmentation of CT/MRI scans was obtained to calculate the baseline and post-shunting/collapse/re-expansion area of lateral ventricles. Shunt valve characteristics and a longitudinal evaluation of the clinical history and treatment outcomes was then performed in a retrospective fashion. To our knowledge, this is the first longitudinal retrospective study to assess the risk factors, clinical presentation and treatment strategy of VC in IIH, integrating clinical data (headaches and other symptoms) with quantifiable and reproducible measurements of both ventricular collapse (present vs. absent) and ventricular area on imaging.

In exploring the risk factors associated with developing ventricular collapse, we adopted a single-step strategy for statistical inference. We performed logistic regression using the Jeffreys prior, also known as penalized logistic regression or the Firth method. This approach outperforms conventional logistic regression, providing less biased estimates in situations with small sample sizes and several unbalanced and highly predictive risk factors. For all analyses, we set the alpha level at 0.05 to indicate statistical significance.

## Methods

### Study design, inclusion criteria and patient population

After obtaining approval from the Institutional Review Board, we performed a retrospective analysis of patients diagnosed with IIH using Friedman’s adapted criteria for IIH and IIH without papilledema that received VPS at Johns Hopkins Hospital [1]. Inclusion criteria for this study: 1) Patients presenting with a diagnosis of IIH and treated for the first time with a VPS at our Institution; 2) VPS implantation between September 2015 and September 2018; 2) minimum follow-up of 1-year post-VPS.

### Variables collected

We retrospectively reviewed the clinical records in Epic (Epic, Verona, WI, USA) and collected demographic data, diagnosis, type/settings of shunt/ASD, dates/types of operations, intraoperative findings, occurrence of shunt failure/revision, treatment modalities, and outcomes at multiple time-points (immediately and at 2/6 months post-operatively). Opening pressures (OPs) on lumbar puncture (LP) before shunting, papilledema, ICP monitoring, CSF leaks were also collected. We then identified patients presenting with symptoms and radiological signs of VC; followed their clinical course and reviewed therapies, outcomes, and radiologic response. Venous stenosis, stenting, and venous pressures were recorded. Patients with VPS implanted at other institutions were excluded from the final analysis.

### Imaging evaluation

**Fig. 1:**
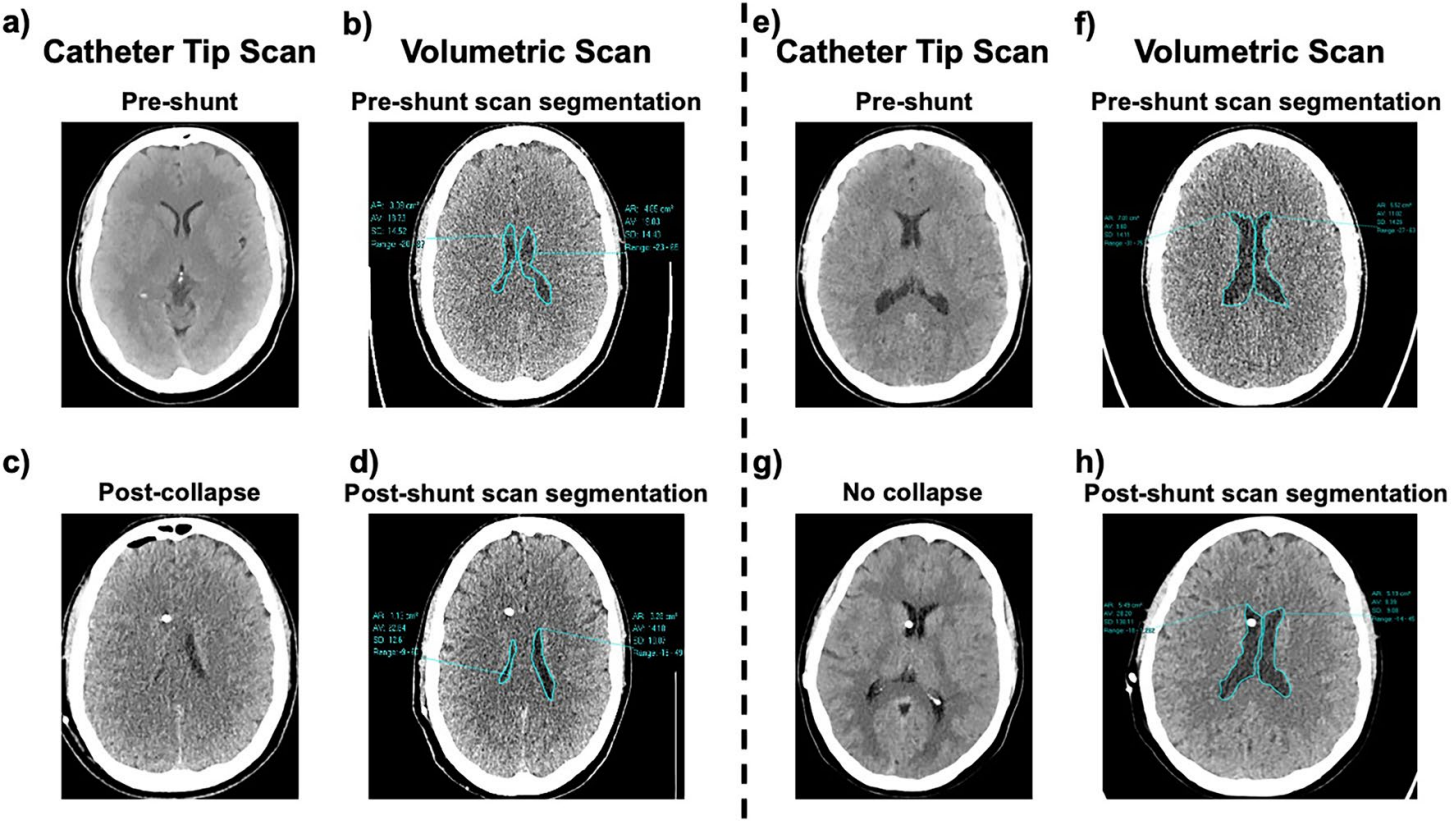
Methods of image segmentation. Fig. 1a–c-e-g) pre- and post-VPS assessment of ventricular patency. In post-VPS scans patency and collapse are evaluated at the level of the catheter tip. Fig. 1b–d-f-h) ventricular area is calculated with manual scan segmentation at the point of maximum width in patent and collapse ventricles

CT/MRI scans were reviewed to identify patients with radiologic VC. Ventricular collapse - VC was defined as absence of CSF around the catheter tip in the shunted ventricle and a normal or negligible volume on the contralateral ventricle (Fig. 1a–h). Preoperative, postoperative, post-VC, and post-treatment scans were also collected for each subject. We then performed manual segmentation and calculated the lateral ventricular area at the point of maximum width for each timepoint (Fig. 1a–h). Importantly, both the occurrence of VC and the radiologic changes in ventricular area were analyzed in relation to the clinical presentation, symptoms, valve type/setting and intervention. Baseline and post-shunting volumes were analyzed as individual risk factors for ventricular collapse. CARESTREAM Vue PACS^®^ software (Carestream, Rochester, NY, USA) was used for image collection, segmentation and analysis. Image segmentation was performed in a blinded fashion by a third investigator, and patient outcomes were not disclosed at the time of analysis.

### Statistical analysis

Collected data were organized in Microsoft Excel (Microsoft Corp., Seattle, Washington, USA). Statistical analysis was performed with RStudio (Boston, MA, USA) and GraphPad Prism^®^ (Version 8.1.0, San Diego, CA, USA) by a third blinded physician. Pearson and Spearman’s tests were applied for assessing correlations. Univariate analyses were performed with Fisher’s exact test, unpaired t-test and Mann-Whitney U-test. The cross-sectional association between ventricular collapse and pre-op volume, post-op volume, BMI, was assessed using bivariate and multivariable generalized logistic regression analyses. Covariates considered in the model were age, gender, opening pressure, Bilateral Sinus Stenosis, BMI, Pre-Shunt Total Ventricular Area, Post-Shunt Total Ventricular Area, Pre/Post-Shunt Area Difference, Symptoms 2- and 6-months post-shunt. P-values < 0.05 were considered statistically significant.

## Results

### Demographic and clinical data

**Fig. 2:**
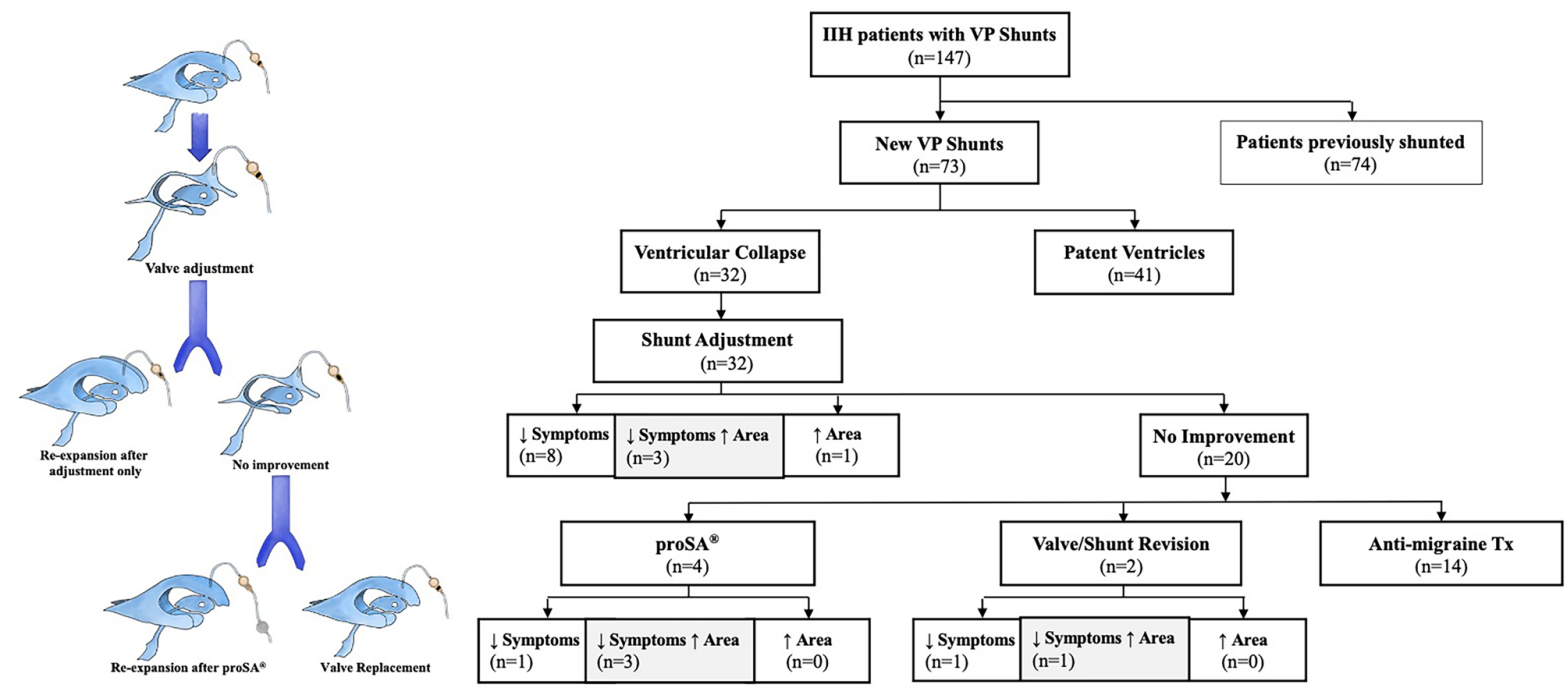
Study workflow and schematic of ventricular collapse timeline

After an initial screening of 147 patients (Table 1; Fig. 2), seventy-three (n=73) treatment-naïve subjects with IIH were identified, including 62 females (86%) and 11 males (14%) (Table 2; Figure 2). Average age among treatment-naïve patients was 39.1 years (SD ± 2.4), mean BMI 38.3 kg/m2 (SD ± 9.2), 26 were previous/current tobacco smokers (26/73, 35%) (Table 2), and mean follow-up from initial shunt implantation (range 12-49) was 28 months. Uni- or bilateral VC on imaging occurred in 32 IIH patients out of 73 (44%) (Table 2).

**Table 1:** Cohort demographics and univariate analyses for individual risk factors of collapse

IIH patients with VP Shunts	All Patients N = 147	Patent Ventricles N = 96 (65%)	Collapsed Ventricles N = 51 (35%)	Univariate Analysis p-value
**Previously Shunted**	**74**	**55** (74%)	**19** (26%)	**0.02†**
**Newly Shunted**	**73**	**41** (56%)	**32** (44%)	
**Age (y)** Previously Shunted Newly Shunted	**40** 41 39.2	**42** 41.4 38.3	**39** 41.5 40.4	0.58 0.21 0.43
**Gender** Previously Shunted Female Male Newly Shunted Female Male	**147** 74 70 4 73 62 11	**96** 47 44 3 41 35 6	**51** 27 26 1 32 27 5	0.40† 0.99† 0.99†
**BMI (SD; kg/m**^**2**^) Previously Shunted Newly Shunted **p-value**	**38.3 ± 9.2** 36.8 ± 9.2 39.7 ± 9 **0.05**	**37.7 ± 8.7** 35.3 ± 8.2 40.7 ± 8.3 **0.003**	**38.8 ± 9.9** 39.2 ± 10.3 38.5 ± 9.8 0.79	0.54 0.08 0.30
**Number of Valves (Average)** Previously Shunted Newly Shunted **p-value**	**250 (1.71/pt.)** 165 (2.3/pt.) 85 (1.2/pt.) ** < 0.001**	**146 (1.69/pt.)** 99 (2.3/pt.) 47 (1.1/pt.) ** < 0.001**	**104 (1.73/pt.)** 66 (2.44/pt.) 38 (1.18/pt.) ** < 0.001**	0.57 0.31

General characteristics of a cohort of 147 patients diagnosed with IIH and shunted with ventriculo-peritoneal shunts at our institution and/or at other institutions before. Chi-square test (†) and Unpaired t-test were used for univariate analysis. Significant p-values are bolded

**Table 2:** Newly-shunted IIH patients, risk factors and univariate analyses for ventricular collapse

IIH patients with New VP Shunts	All Patients n = 73	Patent Ventricles n = 41 (56%)	Collapsed Ventricles n = 32 (44%)	Univariate Analysis p-value
**Pre-shunt variables**				
Age (y)	39.1 ± 2.4	38.3	40.4	0.43
Gender * Female* * Male*	*62 (85%)* *11 (15%)*	*35 (56%)* *6 (44%)*	*27 (84%)* *5 (16%)*	0.99†
BMI (SD; kg/m^2^)	39.7 ± 9	40.7 ± 8.3	38.5 ± 9.8	0.30
Follow-Up Period (range; months)	6–49	6–48	8–49	0.47
Smoking (current and former)	26 (35%)	14 (54%)	12 (46%)	0.99
Bilateral Sinus Stenosis	17 (23%)	11 (27%)	6 (18%)	0.57
Opening Pressure at Baseline (SD)	34.1 (12.2)	35.1 (11.5)	32.6 (13.2)	0.42
Sinus Stenting Before Shunting	4 (80%)	3 (100%)	1 (50%)	
CSF Leak Before Shunting	8 (100%)	2 (100%)	6 (100%)	
Medical Tx^*^	24 (19%)	13 (31%)	11 (40%)	0.99
ICPm	24 (33%)	12 (29%)	12 (37%)	0.43
**Post-shunt variables**				
Sinus Stenting After Shunting	1 (20%)	0 (0%)	1 (50%)	0.99
CSF Leak After Shunting	0 (0%)	0 (0%)	0 (0%)	
Improved Headaches at 2 mos	57%	30 (73%)	12 (68%)	0.17
Improved Headaches at 6 mos	41%	22 (53%)	8 (25%)	**0.02†**
Headaches at 6 mos	31%	9 (22%)	14 (43%)	
Impaired Vision at 6 mos	1.5%	0 (0%)	1 (3%)	
Dizziness-Instability at 6 mos	3%	1 (2.5%)	1 (3%)	
Headaches and Impaired Vision at 6 mos	7%	1 (2.5%)	4 (12.5%)	
Post-Shunt Scans	3.58	3.58	3.59	0.99
Number of Valves * Replaced Valves*	1.2/pt.	45 (1.1/pt.) *4/41 (10%)*	38 (1.18/pt.) *6/32 (18%)*	0.31
Number of Catheter Revisions * Proximal Catheter* * Distal Catheter*	0.7/pt.	4 (10%) *3/41* 1/41	15 (47%) *6/32* 9/32	**0.004**

Demographic and clinical data of 73 IIH patients shunted for the first time at our institution. Right column - univariate analyses calculated with Fisher’s exact test, paired t-test, Mann-Whitney U test and Chi-square test (†). Significant p-values are bolded

### Risk Factors for ventricular collapse

We performed univariate and multivariate analyses on pre- and post-shunting CT/MRI scans to individuate risk-factors linked to VC (Table 3 ; Supplementary Table 1). On univariate analysis, VC was associated with both pre-operative ventricular area (rho = −0.36; *p* = 0.001) and post-operative ventricular area (rho = −0.62; *p* < 0.001) (Table 3). Right and left ventricular area separately, before and after shunting, were also correlated with the occurrence of VC (Table 3).

**Table 3:** Effect of individual risk factors on ventricular collapse upon shunting

Area measurements	Ventricular Collapse
**Pre-Shunt Total Ventricular Area**	**p = 0.001** **rho = −0.36**
Pre-Shunt Left Ventricular Area	**p < 0.001** **rho = −0.44**
Pre-Shunt Right Ventricular Area	**rho = −0.25**
**Post-Shunt Ventricular Area**	**p = 0.0002** **rho = −0.62**
Post-Shunt Left Ventricular Area	**p = 0.001** **rho = −0.49**
Post-Shunt Right Ventricular Area	**p = 0.001** **rho = −0.64**
**Pre/Post-Shunt Area Difference**	p = 0.09 rho = 0.19

Logistic and linear regression analyses. Association of pre-shunt, post-shunt and pre-/post-shunt difference in right, left and total ventricular area with risk of ventricular collapse. Significant p-values are bolded

Importantly, factors thought to play a role in IIH and VC, such as age, gender, BMI, sinus stenosis, OP, and tobacco use were not significantly associated with ventricular collapse on univariate or multivariate analysis.

Two combinations of factors, either predisposing to VC or associated with shunting, were included in a multivariate analysis model Table 5. The first model, a Pre-Shunting combination of Age, gender, OP on LP, bilateral sinus stenosis, BMI and pre-shunt total ventricular area, was associated with ventricular collapse as a regression model (R^2^ = 0.08, *p* = 0.08) without reaching significance. Pre-shunt total ventricular volume was the only significant individual factor (*p* = 0.008). The second model, including only post-Shunting factors, such as post-shunt total ventricular volume, pre/post shunt area difference, and symptoms at 2 and 6 months, was significantly correlated with development of VC (R^2^ = 0.3, *p* < 0.001) (Table 5).

### Symptoms and radiologic findings of ventricular collapse

Thirty-two (32) subjects experienced VC after VPS, 15 of which developed bilateral collapse, while the remaining 17 had unilateral-right VC. Interestingly, VC was found to be significantly associated with headaches, decreased vision and vertigo at 6 months post-shunting (rho = −0.29; *p* = 0.01; Supplementary Table 1), indicating a possible pathophysiological connection with these manifestations. Although common, this association was not always present in our cohort. In eight cases of VC (8/32, 25%), a subjective improvement in headaches and other related manifestations was reported at 6 months post-shunting. Resolution of headaches/vision impairment/vertigo, and the different combinations of these symptoms were considered in our analysis.

A statistically significant decrease in ventricular area post-VPS was also noticed among the subjects that subsequently developed VC, followed by another reduction in ventricular area that led to VC. Specifically, the total area shrank from 10.5 cm^2^ to 6.3 cm^2^ immediately post-VPS (*p* = 0.02; Table 4) versus from 12.5 cm^2^ to 11 cm^2^ in the patent ventricles cohort (*p* = 0.35; Table 4) and from 8.4 cm^2^ to 5.2 cm^2^ in the VC group (*p* < 0.001; Table 4). A further non-significant decrease was noticed after radiologic VC was diagnosed on CT/MRI images, from 5.2 cm^2^ to 4.3 cm^2^ (*p* = 0.08; Table 4).

Interestingly, symptoms are associated not only with collapse, but also more generally with smaller ventricular area (rho = −0.32; *p* = 0.006). A larger difference in ventricular area between baseline and post-VPS scans was also found in patients with VC, compared to patent ventricles. This finding was significant when total area (patent −1.46 cm^2^ vs. collapsed −3.15 cm^2^, *p* = 0.02; Table 4) and right-ventricular area (patent −0.57 cm^2^ vs. collapsed −2.30 cm^2^, *p* < 0.001; Table 4) were analyzed, suggesting that VC is the result of a more pronounced decrease in volume in ventricles already small at baseline.

**Table 4:** Multivariate regression analyses of Pre-Shunting and Post-Shunting Risk Factors for Ventricular Collapse

Independent variables	Ventricular collapse	p-value
Age	0.32	**R**^2^ **= 0.08**
Gender	0.46	**p = 0.08**
Opening pressure	0.74	
Bilateral sinus stenosis	0.51	
BMI	0.12	
Pre-Shunt total ventricular Area	0.008	
Post-Shunt total ventricular Area	<0.001	**R**^2^ **= 0.3**
Pre/Post-Shunt Area Difference	0.02	**p <0.001**
Symptoms 2 months Post-Shunt	0.82	
Symptoms 6 months Post-Shunt	0.14	

Model 1 – Predisposing Variables: association of age, gender, OP, bilateral sinus stenosis, BMI and pre-shunt total ventricular area with risk of ventricular collapse. Model 2 - Post-Shunt Determinants: association of pre-shunt total ventricular area, pre-/post-shunt area difference, symptoms at 2 and 6 months with risk of ventricular collapse. Significant p-values are bolded

**Table 5:** Longitudinal Ventricular Changes in Size at Baseline, Post-Shunting, Post-Collapse and Post-Treatment

IIH patients with New VP Shunts	All Patients n=73	Patent Ventricles n=41 (56%)	Collapsed Ventricles n=32 (44%)	Univariate Analysis p-value
**Pre-Shunting** Area	10.55 cm^2^	12.56 cm^2^	8.43 cm^2^	**0.002**
*Right Ventricle (ipsilateral)*	5.22 cm^2^	5.99 cm^2^	4.39 cm^2^	**0.02**
*Left Ventricle*	5.32 cm^2^	6.56 cm^2^	4.03 cm^2^	0.001
**Post-Shunting** Area	6.32 cm^2^*	11.09 cm^2^	5.28 cm^2^**	**< 0.001**
*Right Ventricle (ipsilateral)*	3.64 cm^2^	5.58 cm^2^	2.09 cm^2^	**< 0.001**
*Left Ventricle*	4.23 cm^2^	5.51 cm^2^	3.18 cm^2^	**< 0.001**
**p-value** (pre- vs. post-shunting)	**0.02**	0.35	**< 0.001**	
**Post-Collapse** Area	n/a	n/a	4.29 cm^2^	n/a
*Right Ventricle (ipsilateral)*			1.61 cm^2^	
*Left Ventricle*			2.68 cm^2^	
**p-value **(post-shunting vs. post-collapse)			0.07	
**Post-Treatment** Area	n/a	n/a	6.29 cm^2^*	n/a
*Right Ventricle (ipsilateral)*			3.66 cm^2^	
*Left Ventricle*			2.69 cm^2^	
**p-value **(post-collapse vs. post-treatment)	n/a	n/a	**0.04**	n/a
**By treatment:**	n/a	n/a		
Shunt Adjustment			**6.03 cm**^2^	0.09†
proSA^®^			**7.11 cm**^2^	**0.004†**
Valve Replacement			**13.32 cm**^2^	n/a
**Area difference by side:**				
**Pre/Post-Shunting** Area	-2.67 cm^2^	-1.46 cm^2^	-3.15 cm^2^	**0.02**
*Right Ventricle (ipsilateral)*	-1.58 cm^2^	-0.57 cm^2^	-2.30 cm^2^	**< 0.001**
*Left Ventricle*	-1.09 cm^2^	-1.18 cm^2^	-0.85 cm^2^	0.40

Ventricular area measurements of 73 IIH patients shunted for the first time at our institution. Right, left and total areas are shown for each timepoint, and patent vs. collapsed ventricles areas were analyzed with paired t-test. Significant p-values are bolded. The effect of shunt adjustment, proSA® addition and valve replacement was also analyzed and compared to pre-treatment ventricular area. † comparison between post-collapse and post-treatment ventricular areas in patients treated with shunt adjustment, ASD addition and valve replacement

### Valve type and setting in ventricular collapse

Valve type and setting were collected and analyzed in relation to the development of VC. Certas^®^ Plus (Codman by Integra, Plainsboro, NJ) programmable valves without Siphonguard^®^ (Codman by Integra) were implanted in 67 patients with newly diagnosed IIH, while six patients were shunted with Certas^®^ Plus with integrated Siphonguard^®^. Certas^®^ Plus valves were set to a resistance level of 4 in one patient, of 5 in 7 other cases, 6 in 64 patients, and 7 in one patient. These settings correspond to OPs of 11cmH_2_O (4), 14.5cmH_2_O (5), 18cmH_2_O (6) and 21.5cmH_2_O (7), respectively. Among patients with VC, 28 had a Certas^®^ Plus set to 6, while only two patients had a setting of 5, one of 7 and one of 4; one had a Certas^®^ Plus with Siphonguard^®^. Furthermore, 4 subjects in the patent group had their obstructed valve replaced, compared to 6/32 in the VC group. Catheter revisions were also more common in the VC group with 15/32 patients undergoing surgery for catheter blockage, compared to 4/41 among the remaining subjects (Table 2).

### The role of opening pressure and postural drainage in the treatment of ventricular collapse

**Fig. 3:**
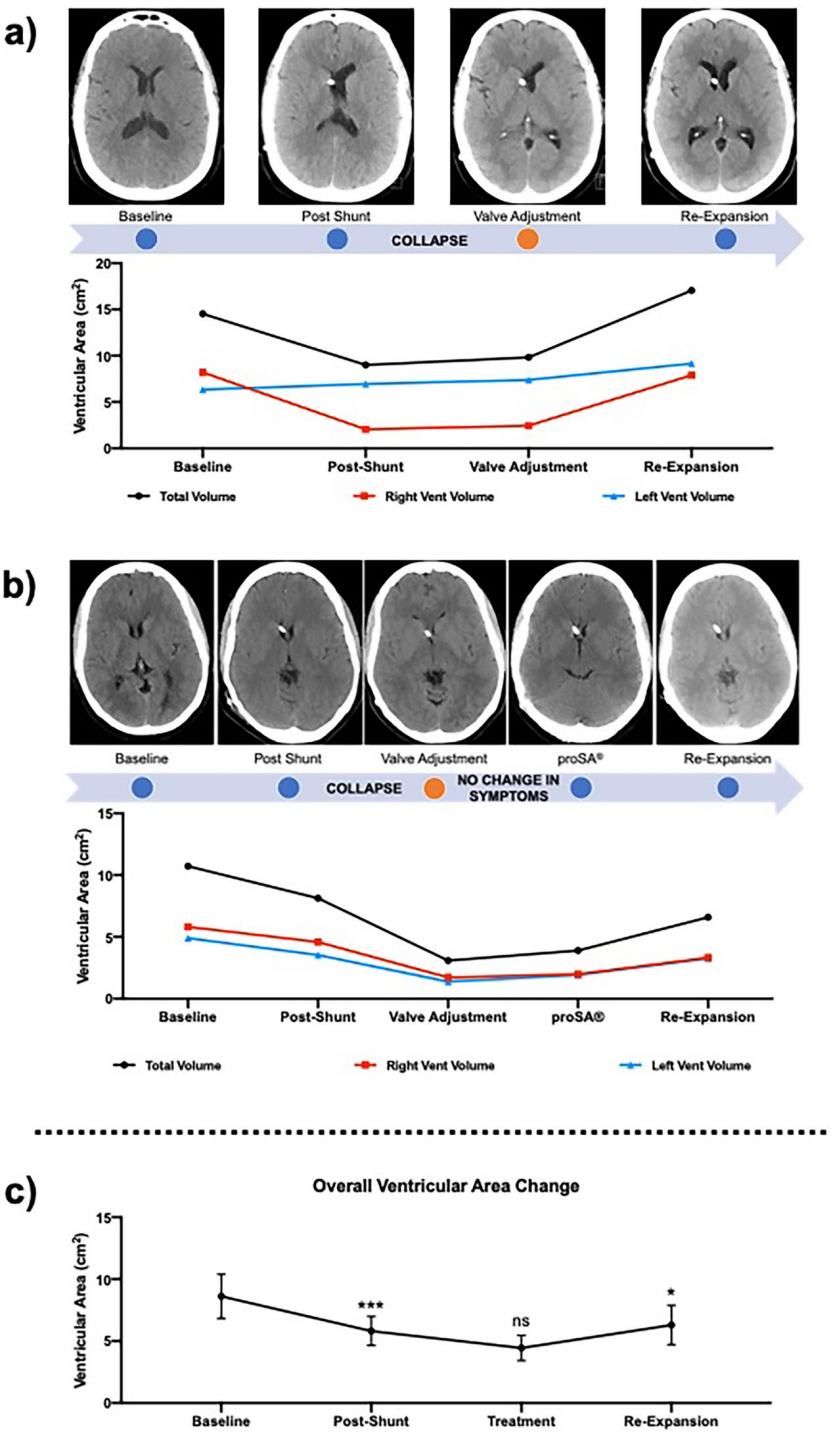
Representative cases of VC treatment. **A**) Example of ventricular re-expansion after valve adjustment in unilateral VC; **b**) the addition of a proSA® ASD achieved ventricular re-expansion and symptomatic improvement in a subject that did not respond to valve adjustment; **c**) average change in ventricular area (with 95% CI) after shunting, post-VC and post-treatment in subjects diagnosed with VC

After VC, symptomatic and radiologic outcomes were analyzed in relation to changes in valve OP, valve replacement, and ASD use (proSA^®^ by Miethke). The first-line measure adopted after radiologic assessment of VC consisted of raising the programmable valve resistance, leading to an increase in the device OP and therefore prompting the lateral ventricles to expand (Fig. 3a, an example of ventricular re-expansion after valve adjustment). Right, left, and total ventricular area significantly increased after valve adjustment in ten patients (10/32, ≃30%), while symptomatic improvement was reported in five subjects (5/32, 15%). Co-occurrence of symptomatic improvement with ventricular enlargement was found only in 5 patients (5/32, 15%). After failure of valve adjustment, the remaining patients underwent either the addition of an ASD, (4/32, 12.5%), valve replacement (2/32, 6%) or migraine therapy. Valve adjustment achieved an average increase in area of 1.75 cm^2^ (SD = 3.08), while the addition of a proSA^®^ achieved a mean ventricular dilation of 3.03 cm^2^ (SD = 0.77) (Fig. 2 ; Fig. 3b, ventricular re-expansion after ASD addition).

Symptomatic outcomes were analyzed in relation to changes in ventricular area on imaging. Specifically, 10/32 patients (≃30%) experienced some degree of isolated ventricular dilation after treatment, and 10/32 (≃30%) noticed an improvement of headaches, confusion, vision, and/or other neurologic complaints. On the other hand, only 9 of 32 patients had evidence of both radiologic and symptomatic improvement (28%).

## Discussion

The introduction of ball-and-spring valves by Nulsen and Spitz in 1949 revolutionized the treatment of hydrocephalus, making conditions with raised ICP surgically treatable [21].

Unfortunately, several complications have arisen in patients with CSF diversion. Shunt “overdrainage” has been recognized as a consequence of supra-physiologic CSF diversion in VPS. This leads to small ventricles on imaging, postural headaches and slow refill of the valve reservoir, as well as other neurologic symptoms [17]. VC can result in Slit-Ventricle Syndrome (SVS), an entity that presents with nausea in the adult population. These findings, although frequent among patients with VPS, have rarely been classified into a well-defined clinical syndrome in adult patients, and the limited available evidence for IIH significantly undermines the efficacy of current treatments.

In this study, we retrospectively investigated a large cohort of IIH patients treated with ventriculo-peritoneal shunts, and analyzed the risk factors, clinical and imaging presentation, treatment outcomes of this approach. IIH patients have several characteristics that make them prone to develop VC upon shunting, among which the most prominent are the slit-like appearance and small volume of ventricles at baseline [14, 15]

Further, raised ICP can determine a significant pressure gradient between the ventricles and the peritoneum, increasing the rate of CSF drainage [22, 23], while the higher distance between the proximal and distal catheters openings might make these devices more prone to overdrainage in adults than in children [24, 25].

### Risk factors of ventricular collapse and their clinical importance

The use of uni- and multivariate analyses revealed that subjects with smaller ventricles at baseline are more prone to undergo uni- or bilateral VC. This finding, although not surprising from a geometric perspective, is relevant in several ways: first, it raises the hypothesis that abnormally small ventricles result from parenchymal turgor, that also provides a greater force for collapse. Secondly, it suggests the potential use of ventricular area on baseline imaging as a surrogate clinical marker to individuate those patients at risk of developing VC upon shunting. Third, smaller baseline ventricles might simply be more prone to collapse after shunting, as less CSF is needed to be drained in order to reduce their volume.

A possible explanation to the different responses to VPS seen in our cohort could be found in brain turgor [26], parenchymal compliance, and the movement of extra- and intracellular water. In support of this hypothesis, the smaller area of lateral ventricles at baseline could be interpreted as a sign of increased parenchymal turgor, reflecting a stronger predisposition to collapse in patients with IIH and reduced ventricular area after VPS. Patients with smaller baseline ventricles experienced a larger shrinkage after shunting, compared to patent ventricles. Patients with VC were found to have a larger decrease in total ventricle area after shunting despite having smaller ventricles pre-procedural (Table 4).

Furthermore, compliance appears to be driven mainly by the displacement and movement of intracranial blood volume, and by its venous component [14, 19, 20, 26]. Similarly, venous congestion and collapse have been linked to cerebral turgor and distensibility by a variety of studies [14, 19, 26], and appear to be involved in the pathogenesis of IIH and SVS. Consistently with this hypothesis, the ventricular area at baseline may be interpreted as a sign of parenchymal turgor, where smaller ventricles correspond to a larger parenchymal elastic modulus and therefore a higher tendency to collapse upon shunting. VPS in fact, by decreasing the ventricular CSF pressure, reduces the vector counteracting parenchymal turgor and promotes collapse. Further, the lower ICP determined by shunt overdrainage has an indirect effect on cranial veins distensibility, making them more prone to collapse in the supine position, and distended when the patient is standing. The contribution of the venous system therefore becomes an important factor in VC pathogenesis and maintenance, both for its effect on craniocerebral compliance and for the bidirectional effects on CSF dynamics [14, 27–31]. Venous congestion and stenosis could impact brain distensibility and intracranial volume, modulating the response to treatment and causing a vicious cycle of collapse and re-expansion. Finally, a negative but important finding is represented by the similar distribution of stenotic venous sinuses recorded in the two cohorts, suggesting that the role played by these factors is likely less relevant than previously thought, and that future studies with more sensitive measures are needed to assess their true contribution to the etiopathogenesis of IIH and VC.

### Effect of valve type and setting on ventricular collapse

Low-pressure valves and shunt overdrainage have been identified as two of the most important risk factors of VC and SVS [18, 31–38]. For this reason, high OP and programmable valves should be useful in treatment along with the addition of antigravity or antisiphon devices. Programmable valves were used in all subjects, while seven patients also received an integrated high-resistance surge-induced alternate high resistance pathway SiphonGuard^®^).

Although the majority of the valves (64 subjects) were set to a high resistance level of 6 (corresponding to an OP of 18cmH_2_O) 28 patients (43% of the total) still developed VC. On the other hand, seven patients (7/73) received a programmable valve with SiphonGuard^®^, and only one experienced VC. These findings, although not generalizable due to the small sample, suggest that despite the use of programmable valves at high settings the occurrence of shunt overdrainage is frequent. The addition of an ASD may therefore improve the flow dynamics and symptomatology.

Further, our evidence suggests that the ability to maintain high ICPs could reduce the risk of VC and maintain the ventricles dilated in the period post-VPS insertion. Although this could cause the persistence of some manifestations of IIH, avoiding the onset of VC might be essential to maintain normal CSF circulation in the ventricular-subarachnoid, interstitial and transependymal spaces. This could also prevent the cycle of collapse and expansion that is so difficult to halt after VC.

### Therapeutic strategies to reverse ventricular collapse

VC is a challenging complication of VPS, usually addressed with an empirical approach, depending on the underlying cause. In some instances, ventricular catheter position is modified or catheters added to drain residual areas of fluid. Unfortunately, this does not address the problem of overdrainage and often results in subsequent collapses. Further, craniospinal disproportion can result in slit and collapsed ventricles and may be best treated with cranial expansion. However, these solutions are not often feasible or first choice of treatment. Valve adjustment appears to be an effective first-line measure in patients with VC, and raising the OP to 21cmH_2_O or higher achieves radiologic or symptomatic relief in approximately ~30% of our cases. In case of failure of this approach, postural overdrainage can be limited by adding an ASD (in our case) to the shunt in use [39–41]. Although the numbers are small in this series and a comparison with medical management of headaches is lacking, all the VC patients that were implanted with an ASD after refractory VC experienced complete radiologic and symptomatic resolution. Despite the cost and mechanical complexity this device might be a valuable addition to consider in cases of refractory IIH or post-shunting VC, and support for this solution can be found in the literature [39–41].

### Association of symptoms and ventricular area after collapse

Shunting is usually effective in improving headaches and papilledema, but these effects can disappear after about six months, both in VC and non-VC subjects (Table 2). Noticeably, unless shunt blockage has occurred, papilledema and ICP remain resolved in patients with the recurrent headaches of IIH, suggesting that other factors might explain shunt-related headaches (Supplementary Table 1).

In our patients that improved after VC only a fraction experienced both symptomatic and radiologic improvement (both headaches relief and ventricular re-expansion). While 10 subjects out of 32 had significantly larger ventricles post-VC resolution on CT/MRI, and 10/32 reported symptomatic improvement, only 9 patients were observed to have both symptomatic and anatomical resolution. It seems clear that the headaches are not directly based on VC and are likely multifactorial. While re-expanding collapsed ventricles may be beneficial from the standpoint of reducing the risk of shunt failure, they are not always associated with the resolution of multifactorial headaches.

### Prevention and treatment of ventricular collapse

VC is generally considered an elusive complication of VPS and its pathophysiologic understanding has frequently been framed in the context of pediatric SVS. Nonetheless, the frequency and severity of VC in adult IIH patients deserves a better comprehension.

Within the appropriate clinical context, a small ventricular area on imaging (under 9 cm^2^ of total area) can be a useful indicator of VC. Such patients may be provided with a system using a higher OP and the addition of an ASD. Based on our series, VC could likely be prevented by the use of a surge-induced high resistance pathway protector (Siphonguard^®^) or adjustable gravity device (ProSa^®^) in the initial shunting of high-risk IIH patients.

Finally, once VC has developed, several measures should be taken to induce ventricular re-expansion. First, the valve OP should be raised to a higher resistance, greater than 20cmH_2_O. This usually allows prompt ventricular re-expansion, despite sometimes resulting in recurrence of IIH “high-pressures” headache symptoms. If this measure fails, the addition of an ASD could reduce postural drainage in presence of symptoms of shunt overdrainage and improve VC.

### Limitations

Our attempt to study the risk factors of VC in IIH and explain their role in determining shunt- and VC-related headaches is not without limitations. First, as a retrospective series performed at a single institution, the design has certain limitations in assessing the role of the main demographic variables on IIH and VC pathogenesis, and future prospective investigation will therefore expand on similar end-points to validate the findings.

The intervals between CT/MRI scans and other instrumental analyses were variable, and ventricular area was estimated with 2D image-segmentation. Significant interpersonal variability in the baseline volume and shape of the ventricular system is present, likely attributable to physiologic anatomical variance. Nonetheless, we considered this methodology a valid approximation of the clinical setting and a first approximation for the study of ventricular collapse. Clearly, a prospective application of multivariate models along with quantitative imaging parameters of ventricular area in the clinical setting, with the aim of better tracking the anatomical response to variable shunt drainage, will allow better prediction and prevention of VC and understanding of the relationships between pathophysiology, shunt function and symptoms.

## Summary and conclusion

Ventricular collapse, often unilateral, was observed in roughly a third of patients after initial shunting, and was associated – together with ventricular area - with worsening symptoms of IIH. This occurred even with a high differential pressure settings of 180 mm water.

High opening pressures and controlled positional drainage are important factors in the treatment of IIH. The observed collapse can be treated by further increasing valve opening pressures alone or with addition of a form of antisiphon device, with resulting re-expanded ventricles and/or reduces symptoms. This is especially true in IIH patients with smaller ventricles, where collapse rates are higher and symptoms redevelop more often. While successful ventricular re-expansion and/or symptoms resolution were achieved in 56% of patients overall, using valve adjustments and additions, these benefits occurred concomitantly in only ~30% of the cases, supporting the idea of a multifactorial etiology for IIH headaches.

In conclusion, our data shows that CSF drainage and valve resistance settings contribute significantly to a high rate of VC symptomatology and clinical progression. Importantly, while VC could be treated with increased opening pressure and gravity regulation, the ventricles volume and symptomatic relief were often not associated. This finding further highlights the importance of CSF overdrainage - rather than mere ventricular area - in the pathogenesis and symptomatology of VC, and suggests a potential explanation for shunt-associated complications.

## Electronic supplementary material

Below is the link to the electronic supplementary material.

Supplementary material 1

## Data Availability

All data and materials included in these studies will be freely shared with any interested party. The data that support the findings of this study are available from the authors.

## Abbreviations

VC: ventricular collapse
IIH: Idiopathic Intracranial Hypertension
CSF: Cerebrospinal Fluid
VPS: Ventriculoperitoneal Shunt
ASD: Anti-Siphon Device
OP: Opening Pressure
LP: lumbar puncture
