# Supplementary material for: A retrospective longitudinal analysis of risk factors, treatment outcomes and imaging parameters of ventricular collapse in idiopathic intracranial hypertension
Source: Fluids Barriers CNS. 2025 Nov 5;22:112. doi: 10.1186/s12987-025-00717-x (PMC12587583; doi:10.1186/s12987-025-00717-x)

**Supplementary Table 1.** **Logistic and linear regression analyses of individual risk factors and their association with VC and Ventricular Size on Imaging**. Main demographic variables before and after shunting associated with the onset of Ventricular Collapse, pre- and post-shunt right, left and total ventricular area. Significant *p-values* are bolded.

| Independent Variable | Ventricular Collapse | pre-shunt Area | post-shunt Area | pre-shunt left-vent Area | pre-shunt Right-vent Area | post-shunt Left-vent Area | post-shunt right-vent Area |
| --- | --- | --- | --- | --- | --- | --- | --- |
| PRE-SHUNT VARIABLES |  |  |  |  |  |  |  |
| Age | p = 0.26  rho = 0.13 | p = 0.26  rho = 0.13 | p = 0.80  rho = -0.03 | p = 0.41  rho = 0.10 | p = 0.17  rho = 0.16 | p = 0.80  rho = 0.02 | p = 0.61  rho = -0.06 |
| Gender | p = 0.90  rho = 0.01 | p = 0.16  rho = 0.16 | p = 0.17  rho = 0.16 | p = 0.26  rho = 0.13 | p = 0.14  rho = 0.17 | p = 0.17  rho = 0.16 | p = 0.13  rho = 0.18 |
| Smoking | p = 0.77  rho = 0.03 | p = 0.59  rho = -0.06 | p = 0.48  rho = -0.08 | p = 0.40  rho = -0.09 | p = 0.75  rho = -0.03 | p = 0.26  rho = -0.13 | p = 0.96  rho = -0.004 |
| BMI | p = 0.38  rho = -0.10 | p = 0.17  rho = -0.16 | p = 0.81  rho = -0.03 | p = 0.40  rho = -0.10 | p = 0.08  rho = -0.21 | p = 0.72  rho = -0.04 | p = 0.71  rho = -0.04 |
| Opening Pressure at Baseline | p = 0.26  rho = -0.14 | p = 0.74  rho = -0.04 | p = 0.84  rho = 0.02 | p = 0.91  rho = 0.01 | p = 0.44  rho = -0.09 | p = 0.71  rho = 0.05 | p = 0.89  rho = 0.01 |
| Papilledema at Baseline | p = 0.44  rho = -0.09 | p = 0.88  rho = 0.17 | p = 0.88  rho = 0.02 | p = 0.82  rho = 0.02 | p = 0.93  rho = 0.009 | p = 0.60  rho = 0.06 | p = 0.76  rho = -0.003 |
| Sinus Stenosis/Hypoplasia | p = 0.34  rho = -0.11 | p = 0.71  rho = -0.04 | p = 0.97  rho = -0.004 | p = 0.76  rho = -0.03 | p = 0.55  rho = -0.07 | p = 0.57  rho = 0.06 | p = 0.65  rho = -0.05 |
| POST-SHUNT VARIABLES |  |  |  |  |  |  |  |
| Symptoms 2 months Post-Shunt | p = 0.52  rho = -0.07 | p = 0.36  rho = -0.10 | p = 0.12  rho = -0.19 | p = 0.24  rho = -0.13 | p = 0.40  rho = -0.09 | p = 0.09  rho = 0.20 | p = 0.24  rho = 0.14 |
| Symptoms 6 months Post-Shunt | **p = 0.01**  **rho = -0.29** | p = 0.12  rho = -0.18 | **p = 0.006**  **rho = -0.32** | p = 0.13  rho = -0.17 | p = 0.15  rho = -0.17 | p = 0.06  rho = 0.22 | **p = 0.002**  **rho = 0.36** |
| Papilledema after Shunting | **p = 0.03**  **rho = -0.24** | p = 0.89  rho = 0.15 | p = 0.22  rho = 0.14 | p = 0.79  rho = -0.03 | p = 0.72  rho = 0.04 | p = 0.30  rho = 0.12 | p = 0.28  rho = 0.13 |
| Sinus Stenting | p = 0.80  rho = 0.03 | p = 0.12  rho = 0.18 | p = 0.39  rho = 0.10 | p = 0.22  rho = 0.14 | p = 0.06  rho = -0.09 | p = 0.13  rho = 0.18 | p = 0.99  rho = 0.001 |

**Supplementary Table 2**

**Classic IIH Diagnostic Criteria**

|  |  |
| --- | --- |
| 1. | **Presence of papilledema** |
| 2. | **Normal neurological exam (excluding cranial nerve 6^th^ palsy)** |
| 3. | **Negative brain imaging (no hydrocephalus, structural lesions or meningeal enhancement); no venous thrombosis** |
| 4. | **Normal CSF profile** |
| 5. | **Elevated opening pressure on lumbar puncture (>25 cmH2O)** |

**Diagnostic Criteria for IIH without Papilledema**

|  |  |
| --- | --- |
| 1. | **Normal neurological exam (excluding cranial nerve 6^th^ palsy)** |
| 2. | **Negative brain imaging (no hydrocephalus, structural lesions or meningeal enhancement); no venous thrombosis** |
| 3. | **Normal CSF profile** |
| 4. | **Elevated opening pressure on lumbar puncture (>25 cmH2O)** |
|  | Criteria 1-4 *plus* Uni- or bilateral cranial nerve 6^th^ palsy |

**Possible** **IIH without Papilledema if 3 of the following neuroimaging findings are present:**

- Empty sella
- Flattening of the posterior aspect of the globe
- Subarachnoid space distention ± tortuous optic nerve
- Stenosis of the transverse venous sinus

**Supplementary Figure 1.** Clinical progression of ventricular volumes in VC treated with individual valve adjustments. 1) pre-VPS volume at baseline; 2) post-VPS volume; 3) ventricular collapse; 4) post-ventricular re-expansion.


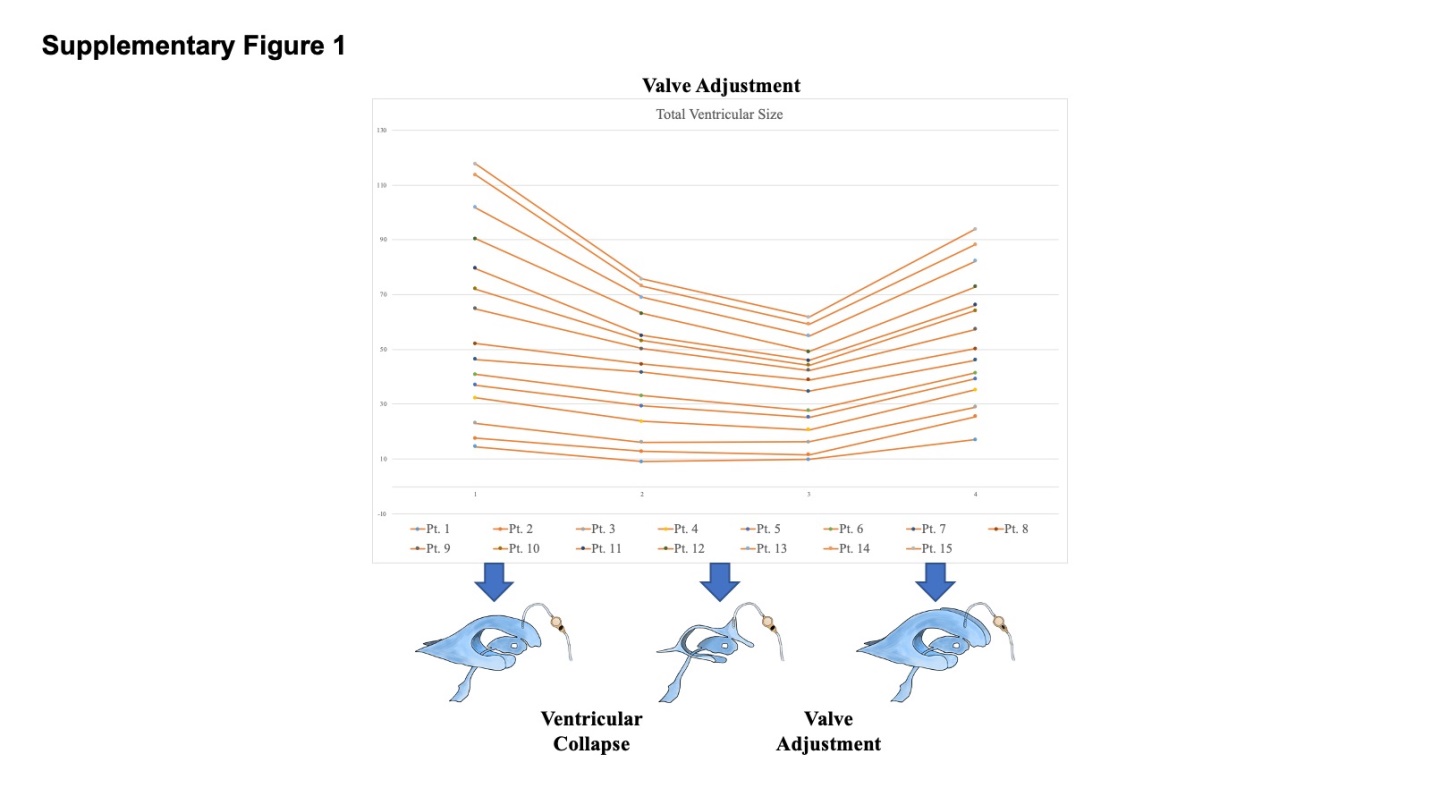


**Supplementary Figure 2.** Clinical progression of ventricular volumes in VC treated with proSA^®^ addition.


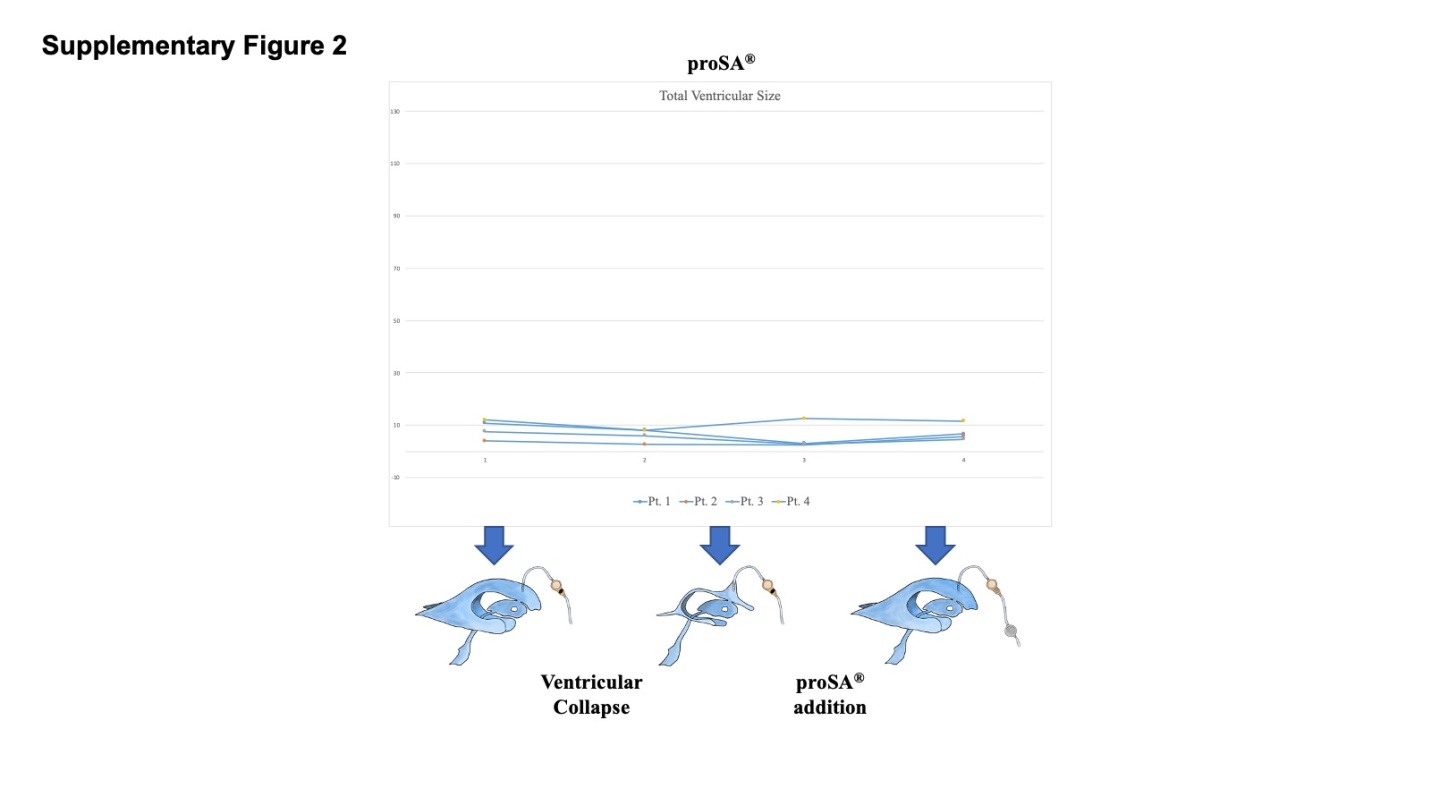


**Supplementary Figure 3.** Clinical progression of ventricular volumes in VC treated with valve replacement.


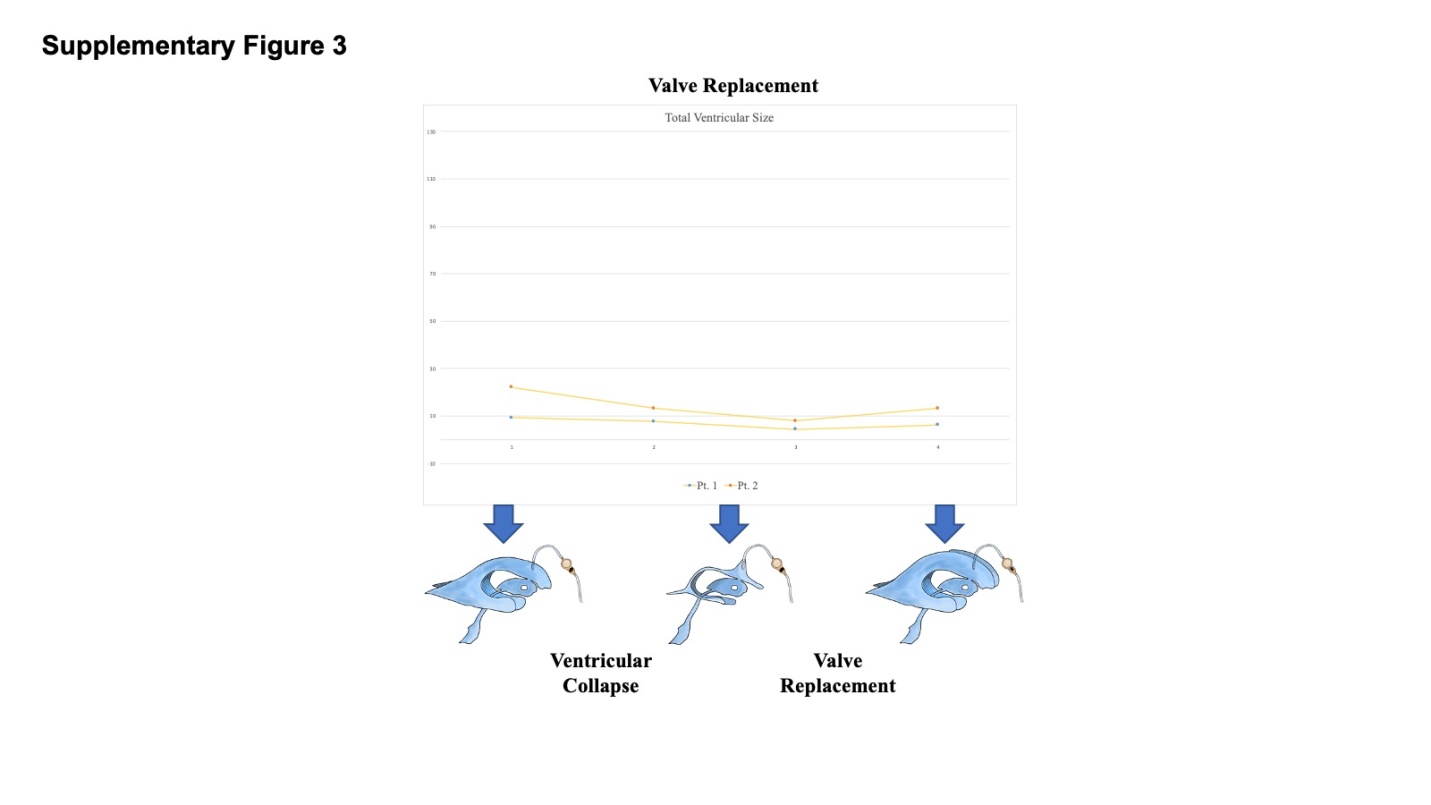

Supplement: Supplementary file 1 — Supplementary material 1 [file 12987_2025_717_MOESM1_ESM.docx]
